# Assessment of anesthetic properties and pain during needleless jet injection anesthesia: a randomized clinical trial

**DOI:** 10.1590/1678-7757-2018-0195

**Published:** 2019-01-14

**Authors:** Allan Carlos Araújo de Oliveira, Klinger de Souza Amorim, Edmundo Marques do Nascimento, Amanda Caroline Batista Duarte, Francisco Carlos Groppo, Wilton Mitsunari Takeshita, Liane Maciel de Almeida Souza

**Affiliations:** 1 Universidade Federal de Sergipe, Departamento de Odontologia, Aracaju, Sergipe, Brasil; 2 Universidade Estadual de Campinas, Faculdade de Odontologia de Piracicaba, Laboratório de Farmacologia, Anestesiologia e Terapêutica, Piracicaba, São Paulo, Brasil

**Keywords:** Anesthesia, dental, Dental pulp, Pain

## Abstract

Pain due to administration of local anesthetics is the primary reason for patients' fear and anxiety, and various methods are used to minimize it. This study aimed to measure the degree of pain during administration of anesthesia and determine the latency time and duration of pulpal anesthesia using two anesthetic methods in the maxilla.

**Materials and Methods:**

A randomized, single-blind, split-mouth clinical trial was conducted with 41 volunteers who required class I restorations in the maxillary first molars. Local anesthesia was administered with a needleless jet injection system (experimental group) or with a carpule syringe (control) using a 30-gauge short needle. The method of anesthesia and laterality of the maxilla were randomized. A pulp electric tester measured the latency time and duration of anesthesia in the second molar. Visual analogue scale (VAS) was used to measure the degree of pain during the anesthetic method. Data were tabulated and then analyzed by a statistician. The t-test was used to analyze the differences between the groups for basal electrical stimulation. Duration of anesthesia and degree of pain were compared using the Mann-Whitney test. A 5% significance level was considered.

**Results:**

There was no statistical difference in the basal electrical stimulation threshold (mA) and degree of pain between the two methods of anesthesia (p>0.05). Latency time was 2 minutes for all subjects. The duration of pulpal anesthesia showed no statistical difference (minutes) between the two methods (p<0.001), with a longer duration for the traditional method of anesthesia (median of 40 minutes).

**Conclusions:**

The two anesthetics methods did not differ concerning the pain experienced during anesthesia. Latency lasted 2 minutes for all subjects; the traditional infiltration anesthesia resulted in a longer anesthetic duration compared with the needleless jet injection.

## Introduction

Fear of pain and anxiety in patients is the most notable reason to avoid dental treatment. Injection of local anesthetics is the most painful phase of a treatment^1^ procedure and a significant reason for its premature discontinuation^2^.

There is a relation between anxiety and fear of pain and the actual sensation of pain. Stress induced by anxiety and fear reduces a patient's pain threshold^3^. Moreover, the sensation of pain further results in increased anxiety, and a cycle is established^1^ ^,^ ^2^.

The efficacy of local anesthetics and the quality standard in needle manufacturing have improved over time. However, the method administrating local anesthetics has practically remained unchanged. Even currently, it is common to use a needle attached to a non-disposable syringe^4^.

Administrating an anesthetic agent with a traditional syringe causes discomfort during the puncture and injection stages^5^. Incorrect handling of the syringe is a determining factor for pain^6^, which is exacerbated due to excessive pressure on the plunger and rapid injection of large volumes of anesthetic solution^7^.

To minimize the painful sensation during local anesthesia, other methods can be adopted, such as applying topical anesthetics prior to injection^8^, using computerized injection systems^9^, manual controlling the injection speed^10^, and using needleless jet injection systems. A needleless system includes a spring coupled to an apparatus that generates sufficient pressure to^11^ push the plunger of the ampoule^12^ and makes the anesthetic solution pass through a micro-orifice at high speed. According to the manufacturer's recommendations, it administers effective local anesthesia with lower anesthetic volumes compared with the traditional anesthesia method^13^.

The absence of a needle in a jet injection can result in a more comfortable experience, as this eliminates the puncture and injection phases^13^, which are considered the most painful steps during traditional anesthesia^5^. This difference is important as approximately one in five adults have phobia of dental anesthesia due to fear of injections, which leads to interruption of dental treatment^14^.

Since there are few studies about the efficacy of jet injection systems in dentistry, the aim of this the clinical trial was to measure and compare the degree of pain, latency times, and pulpal anesthesia duration in both the traditional method of infiltration anesthesia and needleless jet injection during the treatment of maxillary molars, in a split-mouth trial.

## Materials and methods

### Subjects and ethical considerations

**Figure 1:**
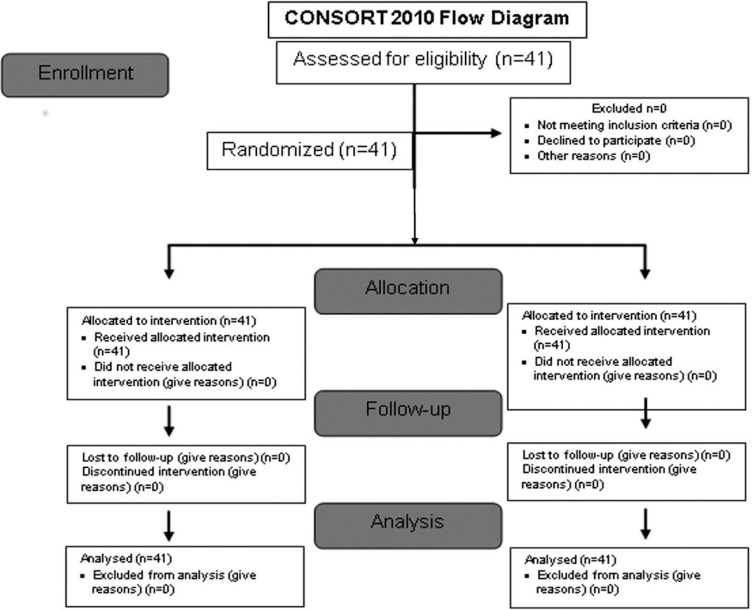
CONSORT flow diagram of patients enrolled in the clinical trial

A randomized, single-blind, split-mouth clinical trial was performed. Experimental design followed the Consolidated Standards of Reporting Trials (CONSORT) guidelines; the experimental flow chart is shown in Figure 1. Our local ethics committee approved the study under the protocol CAAE 62481316.4.0000.5546, and it was registered on the Brazilian Registry of Clinical Trials (RBR-9V37H9). The clinical trial took place from January to November 2017.

Sample-based calculation indicated a requirement for 41 volunteers for an 80% chance of detecting a 10-mm difference in the degree of pain measured by the visual analogue scale (VAS) at a significance level of 5%^15^ ^,^ ^16^. Participants were volunteers who were being treated at the Department of Dentistry of the Federal University of Sergipe, of both genders, with ages between 18 and 40 years, and who required dental restorations in the maxillary first molars with mid-depth class I carious lesions and had healthy maxillary second molars reactive to electric stimulation.

The exclusion criteria were evaluated based on medical history and clinical examination. We excluded individuals with history of allergy or other problems related to any of the components of the anesthetic solution, those with fear of dental treatment, alcoholics and drug users, individuals using analgesics or medications acting on the central nervous system, pregnant women, and individuals undergoing treatment with appliances and orthodontic bands.

All patients signed an informed consent form prior to dental treatment.

### Anesthetic methods

The needleless jet injection method used was the Comfort-in system (Mika Medical; Busan, Korea) and the traditional infiltration anesthesia method was a carpule syringe with a 30-gauge short needle. Lidocaine 2% with epinephrine 1:100,000 (DFL Ind. Com. SA; Rio de Janeiro, Rio de Janeiro, Brazil) was used as anesthetic and the volume was standardized to 1.0 ml for both methods. The anesthetic methods were performed only by an operator that had experience in carrying out both the jet anesthesia technique and oral surgery. For traditional anesthesia, 0.8 ml of the solution of each anesthetic tube was withdrawn with a standard Comfort-in adapter. The carpule syringe (Duflex, S.S. White; Rio de Janeiro, Rio de Janeiro, Brazil) was coupled with a 30-gauge stainless steel short gingival needle (Becton Dickinson; São Paulo, São Paulo, Brazil). The needle was inserted in the muco-vestibular fold above the apex of the maxillary second molars^6^.

Topical anesthetics were not used to avoid interference with the perception of pain^16^. As a result, the total time for anesthetic injection was set at 1.5 minutes because a slow injection decreases the chances of tissue rupture on contact with the anesthetic solution. Consequently, there may have been a reduction in the discomfort experienced during injection^6^.

In the jet method, the anesthetic solution was administered in a fractional manner. Four ampoules were used: the first was filled with 0.1 ml, and the other three were filled 0.3 ml of the anesthetic, as recommended by the manufacturer^13^.

The equipment had a pressurized spring and a silicone cap (recto cap) coupled with the ampoule containing the anesthetic solution for preserving the periodontal tissues. The jet injection system was positioned at 90° in relation to the maxilla with slight compression next to the gingival band inserted at the second maxillary molar. The inserted gingiva was delimited by the mucogingival junction and the coronal (free) gingiva^17^ of the maxillary second molar.

Anesthesia was administered by pressing a button to release the anesthetic solution. The volunteers were informed about the noise produced by the equipment during the release of the anesthetic solution to prevent reflex reactions.

### Outcomes

At the end of each anesthetic method, the pain sensation due to injection was measured using VAS. The volunteer was required to make a vertical line on the 100 mm line, indicating the pain level experienced during anesthesia. A digital caliper measured these values later.

The second maxillary molars were electrically stimulated by the Micro-controlled Digital Pulp Tester (Microeletrônica Indústria e Comércio Ltda-ME; Belo Horizonte, Minas Gerais, Brazil) in the first session to determine the threshold value of the basal electrical stimulation before administrating the anesthetic solution. Only one operator manipulated this device to ensure a standardized protocol.

The pulp electric tester (PET) electrode was positioned on the middle third of the vestibular face of the tooth. At first, the equipment was used with minimum amperage (0 mA), which was gradually increased until the individual reported sensitivity. At this point, the basal threshold value was defined. The maximum amperage used in the study was 80 mA^18^.

Anesthetic latency time: this was defined as the period immediately after injecting the anesthetic to the onset of anesthetic effect. The absence of sensitivity to the electrical stimulus in two cycles of 80 mA confirmed the beginning of the effect and pulpal anesthetic efficacy and defined the latency time measured in 2 and 5 minutes. Anesthesia failure was considered when the volunteer showed sensitivity to electrical stimulus at the tenth minute after anesthetic injection^19^.

Duration of pulpal anesthesia: this corresponded to the period between the beginning of anesthetic action and the moment the tooth returned to its basal electrical stimulation threshold. The maxillary second molars were tested every 10 minutes with the application of 80 mA stimuli until they returned to the baseline threshold value^19^.

### Clinical study protocol

The randomization list was created by the evaluator from the website www.sealedenvelope.com. Randomization was applied to the anesthetic method and the laterality of the maxilla. The split-mouth design resulted in all volunteers undergoing anesthesia using both anesthetic methods (needleless jet injection and traditional infiltration anesthesia). To ensure a blind study, information regarding the randomization was enclosed in an opaque envelope and known only to the operator.

The complete clinical trial protocol required three sessions. After defining the basal electrical stimulation threshold, the volunteer received local anesthesia using either the needleless jet injection or traditional infiltration anesthesia method performed by the operator. The interval between sessions was set at 7 days, and the effect of drug metabolism on pain threshold was excluded^20^.

At the time of anesthesia, the clinician responsible for measuring parameters was not present at the outpatient clinic in accordance with the single-blind study design. In the second minute after anesthetic injection, the PET measured latency time. If the tooth still responded to electrical stimulus, it was stimulated again in the fifth minute after anesthesia.

Glass ionomer cement (Maxxion R® FGM; Joinville, Santa Catarina, Brazil) was used for temporary restorative treatment of the maxillary first molars. It was inserted and accommodated into the clean cavity according to the manufacturer's recommendations, and the process was finished with occlusal adjustment.

The restorative procedure did not exceed 10 minutes. Every 10 minutes, new electrical tests were performed on the maxillary second molar until it returned to the basal electrical stimulation threshold. In this way, the duration of pulpal anesthesia was measured.

In the third clinical session, the volunteers received definitive restorative treatment with an adhesive system (Ambar Universal FGM; Joinville, Santa Catarina, Brazil) and composite resin (Filtek-Z350, 3M-ESPE, São Paulo, São Paulo, Brazil). No adverse effect was observed during the anesthetic procedures.

### Statistical analysis

The numerical variables were analyzed using the Shapiro-Wilk test to verify normality and the Bartlet test to determine the homoscedasticity of their variances The percentage analysis was applied to distribute the genders and choose the anesthetic technique. The t-test was used to analyze the differences between the groups for the basal electrical stimulation threshold values. The duration of anesthesia and degree of pain measured using VAS were compared using the Mann-Whitney test. A 5% significance level was considered for all tests, and they were performed using the BioEstat 5.0 statistical packages (Instituto Mamirauá, Belém, Pará, Brazil) and GraphPad Prism 7.0 (GraphPad software, La Jolla, California, United States).

## Results

**Figure 2:**
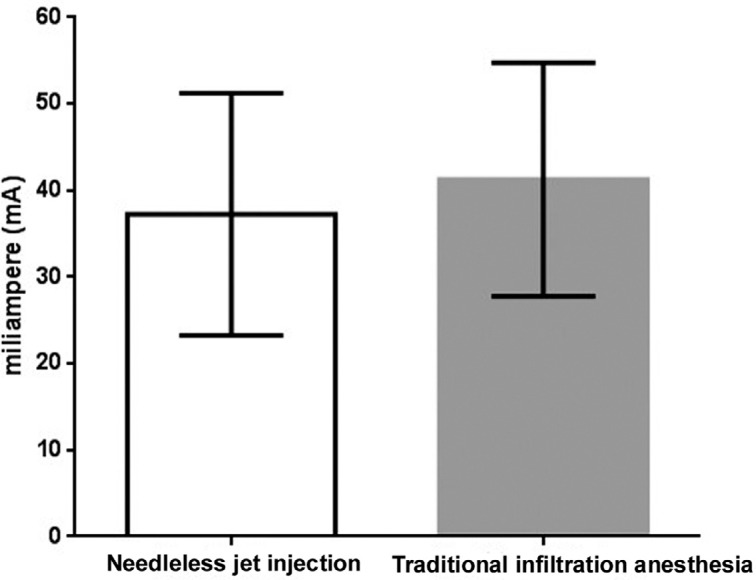
Basal electrical stimulation threshold (mean±standard deviation) as a function of anesthetic method. Student's T test, p>0.05

Of the 41 volunteers, 23 (56.1%) were male and 18 (43.9%) female, and there was no statistical difference (x^2^, p=0.53) between their proportion. The mean age was 25.7 (±4.4) years, which also showed no significant difference (unpaired t-test, p=0.43). According to the t-test, the basal electrical stimulation threshold did not show any statistical difference between its values measured before administration of anesthesia using the two anesthetic methods (p=0.188) (Figure 2).

**Figure 3:**
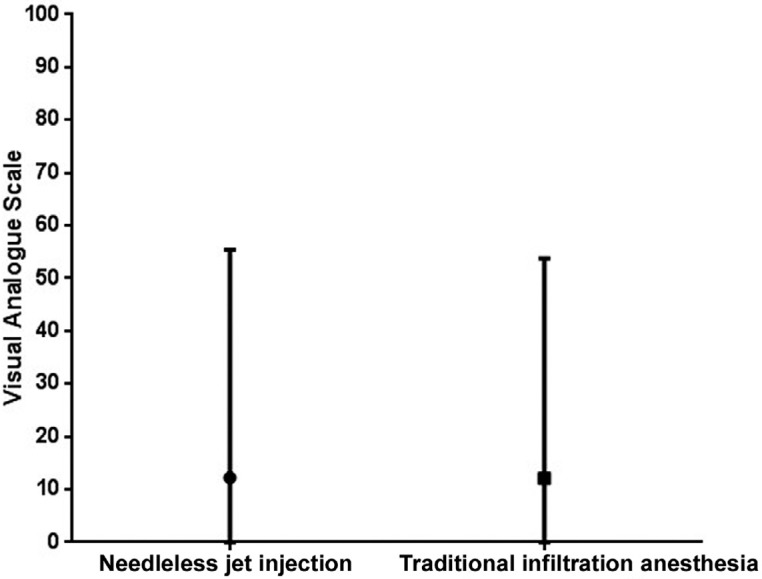
VAS as a function of the degree of pain experienced during administration of anesthesia using the two anesthetic methods in the maxilla. Center bar represents median, maximum, and minimum values. Mann-Whitney test, p>0.05

The Mann-Whitney test showed no statistical difference in the VAS pain during anesthesia (p=0.571) between the two methods. The VAS was 12.2 (0 – 55.4) for the needleless jet method and 12.1 (0 – 53.8) for the traditional infiltration anesthesia (Figure 3).

The latency time recorded for the two anesthetic methods was 2 minutes for all subjects.

**Figure 4:**
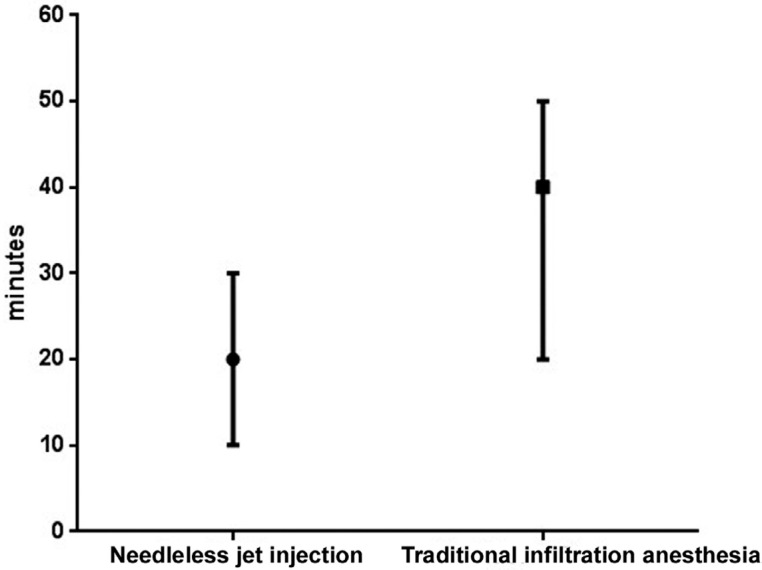
Pulpal anesthesia duration (minutes) for the two anesthetic methods. Center bar represents median, maximum, and minimum values. Mann-Whitney test, p<0.001

The Mann-Whitney test showed a significant difference in pulpal anesthesia duration (minutes) between the two methods (p<0.001). The median duration of pulpal anesthesia for the needleless jet injection and traditional infiltration anesthesia was 20.0 and 40.0 minutes, respectively (Figure 4).

No volunteers required additional anesthesia at any stage of the restorative procedure.

## Discussion

The need for local dental anesthesia should be determined according to the clinical situation and should be administered with minimal pain sensation^21^. Pain during dental anesthesia has a negative impact on the patient. In this study, for both anesthetic methods, the median values were within the score range considered to indicate low degree of pain. This is contrary to the results of a study that compared pain levels during anesthesia between the WAND electronic system and the Injex needleless jet injection system; the mean pain was higher for the needleless jet injection system^22^.

Anesthetic latency and duration of anesthesia are important parameters for planning clinical procedures under local anesthesia. In this study, the determined anesthetic latency time was 2 minutes for both anesthetic methods. This parameter defines the onset speed of anesthetic action and can vary according to the anesthetic agent as well as modifications in anesthetic techniques^23^. Lidocaine has a low dissociation constant (7.7 pKa), resulting in a low anesthetic latency time^6^ ^,^ ^24^ of 2 to 4 minutes^6^. In this study, the aim was not to specifically test the drug's latency, but rather to evaluate anesthetic latency relative to the method of administration, using the needleless jet injection.

Another important finding in this study was that the duration of pulpal anesthesia was lower for the jet injection compared with the traditional syringe injection. This may be attributed to pharmacokinetic processes that take place during tissue diffusion after injecting the anesthetic. The greater is the initial concentration of local anesthetic in the non-ionized form at the injection site, the faster is the diffusion, with unimpeded movement of these liposoluble molecules towards the nerve fascicles in the epineurium^6^.

The local anesthetic diffusion is not unidirectional. A jet injection that deposits the entire anesthetic volume in a fraction of a second possibly allows a higher rate of diffusion by providing a high anesthetic concentration at one time. When it diffuses into the nerve, the local anesthetic becomes progressively more diluted because of extracellular tissue fluids. At this point, nonneural tissues, the capillaries and lymphatic vessels in the region of infiltration, also absorb the drug. As the concentration of extraneural local anesthetic decreases, the concentration of local anesthetic within the nerve rises with progressing diffusion until the concentrations are balanced, and then they begin to reverse. The nerve impulses relaying pain to the brain remain blocked only until the local anesthetic is present in the nerve, and this time period defines the duration of anesthesia^6^.

The possible increase in the speed of all these events due to the high initial concentration of the anesthetic solution after the jet injection compared with the slow infiltration of the anesthetic with a traditional syringe can explain the significant difference in pulpal anesthesia durations.

One clinical trial determined that the mean pulpal anesthetic duration for the traditional technique is 50±9.32 minutes (p>0.001) with a short needle and carpule syringe, whereas the jet injection technique resulted in the lowest duration of 20±3.53 minutes (p>0.001) ^25^. These results are consistent with the findings of our study.

The needleless jet injection eliminates the puncture and needle insertion phases, which may make injection of the anesthetic less painful. However, the pulpal anesthesia duration reported in our study can be considered insufficient for dental procedures such as endodontic treatment and dental extraction. Minor periodontal clinical procedures and class I dental restorations can be satisfactorily performed under pressure anesthesia, provided the treatment is completed within 20 minutes. However, this depends on the skill and experience of the clinician performing the procedure.

The absence of the use of a method to assess the degree of anxiety in individuals prior to anesthesia and the impossibility of a design in which the volunteer remains blind during the administration of anesthesia may be considered as limitations of our study. Moreover, future studies should be conducted to evaluate anesthetic duration with different volumes of lidocaine, other anesthetic drugs, and by applying pressure anesthesia not only the maxilla, but also other to areas of the mandible.

## Conclusions

The two anesthetics methods did not differ concerning pain experienced during the anesthesia. The anesthetic latency was 2 minutes for all subjects, and the traditional infiltration anesthesia resulted in a longer anesthetic duration when compared with the needleless jet injection.
